# Exploring South African Indian men’s understanding of depression

**DOI:** 10.4102/sajpsychiatry.v30i0.2300

**Published:** 2024-10-07

**Authors:** Vashnie Sithambaram, Claire Wagner, Nafisa Cassimjee

**Affiliations:** 1 Department of Psychology, Faculty of Humanities, University of Pretoria, Pretoria, South Africa

**Keywords:** depression, gender roles, societal expectations, stigmatisation, mental health awareness, coping, Indian men, South Africa

## Abstract

**Background:**

Depression is reported as one of the most common mental disorders. Research on Indian men’s understandings of depression is limited.

**Aim:**

The authors aimed to explore South African Indian men’s understanding of depression in a community, and how this guides help-seeking behaviour.

**Setting:**

Community dwelling participants in Gauteng, South Africa.

**Methods:**

An exploratory qualitative design was employed and a purposive sampling method was used to recruit participants. Semi-structured interviews were conducted with seven Indian adult men and analysed using thematic analysis.

**Results:**

The findings of this study yielded a total of six themes. These included understanding of depression, depression is taboo, diverging gender role expectations and depression, help-seeking behaviour, barriers to help-seeking, and mental health community support.

**Conclusion:**

The findings suggest a lack of understanding and awareness of depression among participants and discussions around mental illness being regarded as taboo. Gender roles and societal expectations were considered as one of the contributors to depression onset. Coping and help-seeking behaviour included adaptive and maladaptive coping mechanisms with professional psychological help being least prioritised. Self-stigmatisation and fear of discrimination were highlighted as barriers to help-seeking behaviours.

**Contribution:**

This study contributed to the limited body of knowledge on understanding of depression among Indian men in South Africa and highlighted the importance of mental health awareness campaigns and professional help-seeking behaviour.

## Introduction

Depression is reported as one of the most common mental health disorders^1,2,3^ with the World Health Organization (WHO)^2^ citing that approximately 280 million people worldwide presented with depression in 2023. In South Africa, the prevalence of depression is likely under-reported because of structural barriers in primary health care.^4^ According to reviews and research conducted in the United States of America (USA) and Europe,^5,6,7,8^ depression is under-diagnosed in the male population, and this may be on account of the way men convey their symptoms. Research found that men tend to externalise and somaticise their symptoms.^5^ The diagnostic criteria for depression tend to be skewed towards the clinical presentation of symptoms in women and do not account for the divergence of symptoms displayed by men.^5,6,9^ Under-detection of depression in men stems equally from the understandings both men and their communities have of depression, as well as societal expectations placed on men which may result in a fear of stigmatisation and the failure to seek help.^10,11^

Global reviews on depression in men and help-seeking behaviour indicate limited data reflecting personal perspectives of depression and adaptive coping.^5,7^ Initial evidence from these reviews points to an understanding of depression as taboo and a general discomfort with being diagnosed as depressed. Furthermore, it was found that societal expectations of managing negative emotions in men created challenges to seeking help for depression and compelled them to maladaptive coping such as drug use, emotional isolation and risky behaviours.^5,7^

A myriad of factors underlies the presentation and experience of depression in both men and women. In addition to gender roles, the cultural context is important in how depression is perceived and expressed among individuals.^12^ Culture provides a framework guiding how depression is experienced by individuals, the language they use to communicate symptoms, as well as decisions they make about seeking or refusing treatment.^13^

Cultural backgrounds shape the diagnosis of mental disorders and how they should be managed and treated.^12,14^ In the past, healthcare professionals tended to ignore cultural dynamics and elected to understand and treat people in a similar way following westernised interpretations of mental disorders such as depression.^4,15,16,17^

Despite the plethora of published works of research on cultural components of depression (e.g. ^15,18,19,20,21,22,23,24,25^), studies on Indian men are limited.^9,26,27,28^ Existing research focuses mostly on women.^29,30^ The limited research on Indian men’s experiences of depression^7,9,31^ does not address how depression is understood within the South African Indian male population. The aim of this article is to explore how Indian adult men in a South African community understand depression and how this guides their help-seeking behaviour.

## Research methods and design

An exploratory qualitative design was employed.^32,33,34^ Semi-structured interviews yielded data on participants’ understandings of depression and how this guides their help-seeking behaviour.

Two methods were used to recruit participants. Firstly, pamphlets that contained information about the aim of the study, type of participants sought and the interviewer’s contact details were distributed in public areas such as shopping malls in historically demarcated Indian communities in the Gauteng province in South Africa. Secondly, awareness of the study was created on social media platforms such as Facebook. The majority of the participants were recruited via this medium. Individuals who contacted the researcher were informed with regards to the aim and rationale of the study as well as their rights and responsibilities as study participants.

A purposive sampling method was used to recruit participants with the following criteria:

- adult men (above the age of 25 years)
- self-identify as South African Indian
- resident in Gauteng.

Seven participants were recruited and interviewed. The sample size lends itself to the in-depth analysis required in qualitative studies. As a member of the community that the participants were recruited from, the insider position of the first author allowed participants to feel comfortable sharing their experiences of depression among Indian men. Juxtaposed with the insider position occupied by the first author was her position as an outsider occupying a female role that at times required probing to expand on responses that were vague and unfamiliar.

### Data collection

Interviews took place in February and March 2022 following the recruitment process. Each interview lasted between 40 and 60 min and was audio recorded following consent given by participants. Interviews took place virtually (Zoom or Google Meet) for the safety of the participants and the interviewer because of the coronavirus disease 2019 (COVID-19) pandemic. Interviews were conducted in English and transcribed verbatim.

### Interview guide

The interview guide used in the study was informed by the research aims and literature on male depression with questions focusing on specific topics that were prepared prior to the interview.^35^ These topics included the participant’s understanding of depression, gaining insight into which social structures contribute to their understanding of depression, and what they perceived to be gender disparities with regards to depression. Participants were further required to reflect on their understanding of what caused depression. Particular attention was given to their opinion on whether or not they would seek professional psychological help for depression, and if not, what the alternate ways of coping would be. Lastly, participants were asked what ideal community support would look like for them, and how awareness of depression and professional psychological help could be beneficial to Indian men.

### Data analysis

Once the interviews were transcribed verbatim, a thematic analysis was used to analyse the data. An inductive approach was taken where the themes that were elicited on a latent level were data-driven.^36^ The analysis process began firstly, with repetitive reading of the transcripts and searching for patterns and meaning; secondly, codes were generated from the content and subsequently organised into meaningful categories. Themes were identified by sorting and collating all the relevant coded data and extracts, labelled and a meaning attached with a detailed description. Trustworthiness of the process was ensured through discussions among the researchers, and themes were refined accordingly.^37^ The final analysis was sent to participants to verify the interpretations.

### Ethical considerations

The study commenced once permission was granted. Ethical clearance to conduct this study was obtained from the University of Pretoria, Faculty of Humanities Research Ethics Committee (No. HUM041/0221). Suitable participants showing interest received a detailed information sheet and signed a consent form prior to their interview commencing. To safeguard participants’ identities and maintain confidentiality, each participant was allocated a number (i.e. P1, P2, P3–P7) in place of identifying information.^38^

## Results

The seven participants ranged between the ages of 28 years and 48 years. Two of the participants were married, five were unmarried, and only one did not have a tertiary qualification. All participants were employed in the corporate sector.

Six themes were generated from the analysis: understanding of depression, depression is taboo, diverging gender role expectations and depression, help-seeking behaviour, barriers to help-seeking, and mental health community support. Table 1 provides a list of the themes and sub-themes where relevant.

**TABLE 1:** List of themes.

Theme number	Theme name	Sub-theme name
1	Understanding of depression	1.1 Community perspectives on depression 1.2 Individual perspectives on depression
2	Depression is taboo	-
3	Diverging gender role expectations and depression	3.1 Societal expectations and depression 3.2 Gender and depression
4	Help-seeking behaviour	-
5	Barriers to help-seeking	5.1 Self-stigmatisation 5.2 Fear of discrimination
6	Mental health community support	-

### Theme 1: Understanding of depression

Under theme 1, participants reflected on their understanding of depression from a community perspective (sub-theme 1.1) and an individual perspective (sub-theme 1.2).

#### Sub-theme 1.1: Community perspectives on depression

Participants perceived a general lack in understanding of depression in the community they live in. They reported that during childhood, there was little communication about topics of mental health in their families and further emphasised the ubiquitous belief that mental illness did not exist:

‘So, for example in our community we live in. I would think most Indian people would brush the depression off with, ‘You know what, there’s something wrong with you’, or they don’t take it as seriously as they should. I think maybe there’s a lack of understanding or education on what depression is. But I feel it is taken very lightly in our community.’ (P4, 30 years, single)

According to the participants, this lack of communication and beliefs about mental illness emerge from the lack of knowledge they perceive their communities to have of mental illness especially in relation to the men of the community. One of the participants expressed this as follows:

‘Uhm, to be honest, I would say firstly their knowledge, if I had to rate it from 1-10, it’s probably a one in terms of knowledge and understanding.’ (P1, 35 years, married)

#### Sub-theme 1.2: Individual perspectives of depression

Although participants reported having a basic understanding of what depression is, because of their community’s perspectives they expressed their own understandings of depression in various ways. For example, Participant 1 viewed depression as patterns of negative thinking as well as the inability to think positively:

‘Almost always want to see the bad in every situation. So, it’s again, just tough for them to be optimistic and positive about situations.’ (P1, 35 years, married)

Participant 2 regarded depression to be a ‘mental block’ to people being themselves or what participant 6 sees as ‘being the normal person that they are’.

Four of the participants revealed that depression caused the individual to feel that all areas of life were ‘beyond your control’ including their work and relationships. Some participants considered biological changes in the brain as the cause of negative emotions that are also beyond the control of a depressed individual.

Despite participants’ views on limited community understandings of depression, their exposure to tertiary education, work environments and social media that promoted mental health provided them with a broader understanding of what depression is and how to seek help. For example, Participant 4 felt that he had:

‘[*A*] slightly greater understanding of depression’ as he was aware of the ‘channels that one would seek out in the event that you need further guidance.’ (P4, 30 years, single)

### Theme 2: Depression is taboo

Participants experienced hostility when they expressed concerns about their mental health. They were forced to pretend and not voice their suffering:

‘[*N*]obody is experiencing it.’ [*and*] ‘[*I*]f it’s something in someone’s mind, they need to get over it.’ (P6, 39 years, single)

Participant 7 expresses this as follows:

‘Oh, it’s actually taboo, I think in the broader side of it; it’s like we, we are not allowed to say that we are depressed, we are not allowed to sit down and talk about it because it’s not seen as it being serious, in our community, you know, it’s – I think – it’s like you know, ‘Oh, you could be overreacting, just get along with it.’ (P7, 32 years, single)

### Theme 3: Diverging gender role expectations and depression

Two sub-themes related to the role of societal expectations (sub-theme 3.1) and gender in depression (sub-theme 3.2) were generated.

#### Sub-theme 3.1: Societal expectations and depression

The participants experienced expectations of having to achieve while simultaneously putting on a brave face and not being affected by external stressors. These pressures included financial and/or materialistic demands and marital expectations. Indian men in the participants’ communities are expected to be stoic in all areas of their lives as a sign of masculinity and strength:

‘If you are a boy, and you get into a fight, you need to pick up your fists and fight back … it is these kinds of things that the Indian community is teaching the young, that there’s basically a gender expectation that if you are male, you need to be strong.’ (P4, 30 years, single)

‘An Indian guy just takes care of himself and when he gets married, he has to take care of his wife, he needs to take care of his wife’s family, he needs to take care of his wife still, his wife’s sisters, everybody becomes some responsibility, and that becomes something that you know, you need to be a strong man.’ (P6, 39 years, single)

Participants are afraid to voice their need for assistance as this would place them in a position of ‘disappointing their families’ (P3). Participants felt that gender role expectations placed a lot of pressure on Indian men as their success as a son is interlinked with the success of their family which is reflected in ‘unsupportive’ (P7) familial conversation.

#### Sub-theme 3.2: Gender and depression

Participants’ perceptions about gender and depression showed that women were more expressive in the intensity of their emotions than men and were more likely to ‘break down’ (P6) than men with depression:

‘They also show signs of a lack of interest … they would like to open up with their feelings, it’s socially accepted that you know, that a woman would feel hurt. Whereas a man should just rather deal with his emotions and carry on.’ (P4, 30 years, single)

In spite of these differences, four of the seven participants perceived women’s behaviour as similar to men when depressed with symptoms such as withdrawal from social activities and isolation.

### Theme 4: Help-seeking behaviour

Six of the seven participants reported the belief that seeking professional psychological help was beneficial when experiencing distress:

‘I personally feel that it’s important to seek help when it’s early or when you first realise that, you know, something is wrong, leaving something for long only causes more issues.’ (P4, 30 years, single)

‘… I do believe that it’s necessary and probably because it is something that is a mental issue, so the only way you can fix it is by getting professional help.’ (P1, 35 years, married)

Contrary to the foregoing views, Participant 5 reported that his views towards help-seeking for depression were shaped by his ‘strict’ upbringing on the non-existence of mental illness: ‘you go on with life … brush it under the carpet’ (P5) which may indicate coping by suppressing difficult emotions.

Even though they were in support of professional help, six of the seven participants stated that they were more likely to consult a friend, family member, spouse and/or partner, religious leader, doctor before approaching a psychologist:

‘I’ll speak to someone who I’m extremely close to … I think they make you feel comfortable speaking about it. You won’t feel afraid.’ (P3, 31 years, single)

Participant 6 described how speaking to other men who have experienced sadness in their lives brought more comfort than consulting a professional person who they might not be able to relate to:

‘And the only time he breaks down is by himself with his guys. I’ve experienced that with just friends, males being around males, and when there’s a few drinks start going, the truth and the actual sad side of their lives come out. I feel a little bit more comfortable talking to another guy who has experienced that than speaking to a female. Sometimes, I just feel like you need that real person that’s next to you. That has gone through this. Not has gone through from a textbook point of view but one who has actually gone through real world stuff, and can relate, rather than just sitting there with a checkbox and seeing if you’re hitting the right buzzwords, are you saying the right things, or if your eyes are doing the right things or wrong things. Rather, someone who listened.’ (P6, 39 years, single)

Other coping behaviours included finding a hobby, embracing religion, substance abuse, excessive exercise and financial mismanagement.

### Theme 5: Barriers to help-seeking

Participants spoke of two barriers to help-seeking: self-stigmatisation (sub-theme 5.1) and the fear of discrimination (sub-theme 5.2).

#### Sub-theme 5.1: Self-stigmatisation

Fear of stigmatisation linked to their perception of depression may prevent some Indian men from seeking professional psychological help. According to a participant:

‘Feel like less of a man. You know, things like that there. That masculinity thing plays a part to try and avoid it. Let me keep my manhood by not talking about it. But instead, I want to say something. I don’t want to deal with judgment.’ (P3, 31 years, single)

#### Sub-theme 5.2: Fear of discrimination

Participants feared expressing their emotions and being vulnerable may ostracise them from their community, friends and loved ones while wanting to feel safe discussing their mental health:

‘It means that I need to feel safe enough to go up to anyone in my community and actually talk about it … when I speak, I don’t want to have to feel judged … I don’t want someone to think of me as if something is wrong with me. I would want the other person to engage with me and listen to me and ask me questions and try to understand and, as I said, to feel accepted.’ (P1, 35 years, married)

Another participant echoes this fear of judgement by others for going to therapy and also the uncertainty of explaining the reasons for going to a therapist, to work colleagues or significant others:

‘I think it’s the stigma, the stigma attached to you know, who’s gonna, who’s gonna see me, who’s going to basically see me going out to a psychologist, you go through to the psychologist, what if the psychologist is going to tell me, you know? Is it really confidential? What explanation am I going to give to the next person?’ (P4, 30 years, single)

### Theme 6: Mental health community support

Participants advocated for introducing mental health awareness and help-seeking mechanisms from a young age with campaigns and psycho-education in school curricula.

‘Start instilling values from a little age that it’s okay to deal, it’s okay to be, it’s okay to deal with certain stages in your life, it’s okay to deal with depression. It’s basically okay for men and women to chat or to talk openly about the way that they feel. In terms of the community showing support, maybe there should be more support initiatives to advertise something in the paper that focuses more on the emphasis on mental health. Do you know, are you feeling depressed? Do you want to talk to somebody? Reach out here, have a social gathering.’ (P4, 30 years, single)

This may assist children to understand that boys can also be vulnerable and empower men and women to express their emotions without the fear of discrimination:

‘It means that I need to feel safe enough to go up to anyone in my community and actually talk about it … when I speak, I don’t want to have to feel judged … I don’t want someone to think of me as if something is wrong with me. I would want the other person to engage with me and listen to me and ask me questions and try to understand and, as I said, to feel accepted.’ (P1, 35 years, married)

Participants suggested regular awareness campaigns, pop-up counselling sessions and advertisements (on traditional platforms such as billboards as well as social media platforms) to increase community knowledge about mental health and where to seek help.

## Discussion

Our study aimed to explore a community of South African Indian men’s understanding of depression and help-seeking behaviour. Six themes described their understanding of depression as embedded in their community’s perspectives and the role of societal expectations of men in dealing with mental health and ways of seeking help. Participants who reported having some understanding of depression attributed their knowledge to the contexts (i.e. tertiary education, the workplace and social media) that encouraged mental health awareness. This speaks to the various forms of personal exposure and interpersonal interactions with society that assisted in how participants perceived depression.

Cultural expectations placed on Indian men by their families was regarded by participants as one of the main reasons for depression onset. These demands included being compared to other members of their families and being under constant pressure to perform and succeed in all areas of life.^26^ Participants’ talk about the expectations they experienced, evoked historical gender, social and cultural roles using words such as ‘strong’, ‘provider’, ‘leadership’, ‘success’ and ‘achievement’ among others. These roles are imprinted on men from birth, directing socially appropriate behaviour, responses and interactions. Following prescribed gender roles about masculinity is a guarantee for success as a man.^10^

Although mental illness in Indian communities is frequently connected to ill will, black magic, witchcraft, the evil eye or spiritual possession,^23,39^ when asked their view, participants did not mention spiritual causes of mental illness as being significant. Instead, they spoke about the role religion played in helping them to cope with mental issues. Religion has also, however, been implicated in encouraging gender differences and placing expectations on men. The prevalence of social media and engagement in tertiary education may have exposed participants to topics of mental health. Through their personal experiences, participants spoke of greater knowledge which allows for alternative perspectives that may differ from their cultural beliefs about mental illness.

Depression was viewed as taboo or non-existent by participants because of their lack of knowledge and understanding of mental illness. Furthermore, even though mental health is discussed openly in work and educational environments, participants are unable to have conversations about depression with their loved ones or friends.

This may lead to alternative coping methods with responses from participants highlighting adaptive and maladaptive strategies. Maladaptive coping mechanisms mentioned by participants included the excessive use of alcohol and what participants described as an unhealthy amount of physical exercise leading to isolation and exacerbation of possible depressive symptoms. Adaptive coping mechanisms indicated by participants included talking to their significant others, pursuing a hobby and participating in religious acts.

Interestingly, participants considered psychological support as a last resort because of fear of stigmatisation and discrimination. An increase in information and access to knowledge about mental health has not transformed some cultural-specific societal norms and belief systems resulting in a failure to meet the needs of Indian men as described by the participants.^9^ The understanding of depression among Indian men in some South African communities as taboo or non-existent may persist and consequently discourage their help-seeking behaviour for depression.

## Limitations of the study

The small purposive sample of seven participants with a limited age range and geographical location restricts the generalisation of findings to the larger South African Indian male population. Including a more diverse sample in future studies may broaden our understanding of depression in communities.

## Recommendations

It is recommended that awareness campaigns focused on culturally specific understandings of mental health be prioritised. Collaboration with mental healthcare practitioners who provide psycho-education to address and manage mental illness such as depression may lessen the stigma and facilitate help-seeking behaviour. The advantages of mental healthcare could address maladaptive coping mechanisms that may exacerbate symptoms of depression.

## Conclusion

Six themes generated from this study revealed that participants viewed their communities as having a limited understanding of depression, where it is often considered a taboo topic. Symptoms of depression may not be recognised by participants should they themselves or someone they know be experiencing it.

Furthermore, gender role expectations may precipitate depressive symptoms. With regard to coping with depressive symptoms, seeking psychological help was not a priority; instead, participants listed adaptive and maladaptive alternative coping mechanisms. Reluctance to seek help was attributed to the fear of discrimination. This study contributed to the limited body of knowledge on the understanding of depression among Indian men in South Africa and highlighted the importance of mental health awareness campaigns and facilitating professional help-seeking behaviour.
